# Fentanyl Induces Behavioral Sensitization and Decreases Class IIa HDAC Expression-Activity in Brain as Measured by [^18^F]TFAHA PET Imaging in Female and Male Rats

**DOI:** 10.3390/brainsci16070684

**Published:** 2026-06-29

**Authors:** Cameron J. Davidson, Itzick Nahmoud, Mahmoud Teran, Erek Binkowski, Nareen Sadik, Majd A. Yahya, Susanne Brummelte, Alana C. Conti, Nerissa T. Viola, Srinivasu Kallakuri, Shane A. Perrine

**Affiliations:** 1 Foundational Medical Studies, Oakland University William Beaumont School of Medicine, Rochester, MI 48309, USA; cjdavidson@oakland.edu; 2 Department of Psychiatry and Behavioral Neurosciences, Wayne State University School of Medicine, Detroit, MI 48202, USA; itzick.nahmoud@med.wayne.edu (I.N.); mteran@oakland.edu (M.T.); hr6178@wayne.edu (E.B.); majyahya@med.umich.edu (M.A.Y.); aconti@med.wayne.edu (A.C.C.); skallakuri@wayne.edu (S.K.); 3 Translational Neuroscience Program, Wayne State University School of Medicine, Detroit, MI 48202, USA; sbrummelte@wayne.edu; 4 Department of Psychology, Wayne State University, Detroit, MI 48202, USA; 5 Department of Oncology, Karmanos Cancer Institute, Wayne State University School of Medicine, Detroit, MI 48202, USA; nviola@wayne.edu

**Keywords:** fentanyl, locomotor activity, behavioral sensitization, sex differences, opioids, epigenetic changes, class IIa HDAC, rat, PET

## Abstract

Background: Although fentanyl significantly contributes to opioid-related morbidity and mortality, little is known about the epigenetic changes that may influence long-term neuronal adaptations. Objective: The effects of repeated fentanyl administration on class IIa histone deacetylase (HDAC) expression-activity were studied using the radiotracer [^18^F]TFAHA and positron emission tomography (PET) imaging in a model of fentanyl-induced behavioral sensitization. Methods: Female and male Wistar rats received 14 days of fentanyl (20 μg/kg) or saline injections and a 14-day drug-free period followed by a single fentanyl or saline challenge dose on day 28. Locomotor activity (LMA) was measured on days 0, 1, 14, and 28 with PET imaging being performed at baseline and again on day 28 following the fentanyl/saline challenge and LMA. The percent change in standard uptake value (body weight corrected) between pre- and post-administration was calculated as a measure of class IIa HDAC expression-activity. Results: Repeated fentanyl exposure resulted in significantly increased LMA in both sexes compared to controls. Females displayed an earlier onset (day 1) and a greater magnitude of behavioral sensitization on days 14 and 28 compared to males. Fentanyl significantly decreased class IIa HDAC expression-activity across time in the whole brain and in reward-related brain regions without sex differences. Conclusions: Prolonged fentanyl exposure induces robust sex-specific locomotor sensitization with varying magnitude over time, suggesting differential neuroadaptive processes. Fentanyl also appears to induce epigenetic changes in the brain independent of sex and region. The effect of fentanyl on class II HDACs may not directly impact the expression of behavioral sensitization.

## 1. Introduction

Fentanyl is a synthetic μ-opioid receptor agonist, and when used properly, fentanyl is an excellent pain therapeutic and preanesthetic agent. However, when used illicitly, the rapid onset of action, high potency, short duration, and likelihood of being used as a contaminant within other substances contribute to the high rates of fentanyl use, increasing diagnosis of Opioid Use Disorder (OUD), and high risk of overdose mortality. In fact, fentanyl is the main culprit for the third and fourth waves of the opioid epidemic [1,2]. The brain circuitry involved in fentanyl use as well as many other substances of abuse is relatively well known [3,4,5,6,7]; however, less is known about the impact of fentanyl on epigenetic regulation. Therefore, it is critical to gain an understanding of the long-term neurobiological adaptations that arise following the chronic fentanyl exposure, abstinence, and relapse that underlie the persistence of OUD, including the behavioral manifestations of dependence, tolerance, and sensitization.

Behavioral sensitization to drugs of abuse in rodents is a well-studied phenomenon where repeated drug exposure causes progressively greater and enduring behavioral responses, commonly measured by locomotor activity (LMA) in an open field [8,9,10,11]. Studies have shown that the long-term neuroadaptations underlying behavioral sensitization occur in the brain’s reward circuitry, namely the mesolimbic dopamine pathway from the ventral tegmental area (VTA) to nucleus accumbens (NAC) and regulatory regions such as the medial prefrontal cortex (mPFC) and hippocampus (HPC) [10,12]. However, the brain mechanisms underlying fentanyl-induced behavioral sensitization are not well understood. Importantly, we have shown that fentanyl produces greater LMA and behavioral sensitization in female rats compared to males [13]. Therefore, fentanyl-induced LMA and behavioral sensitization provide a robust and translatable model to measure the region-specific neural effects of fentanyl and probe epigenetic mechanisms that may underlie observed sex-specific effects.

Several studies support the role of epigenetics, including histone and chromatin modifications that regulate gene transcription, in brain regions and circuits underlying drug-taking and other drug-related behaviors, including behavioral sensitization [14,15,16,17,18]. Class IIa histone deacetylases (HDACs) are postulated to exert repressive activity partially via recruitment of class I HDACs [19] and are thought to act as signal transducers by transporting between the nucleus and cytoplasm [20]. Within the class IIa HDACs, HDAC4 and HDAC5 appear to play complex neurobiological roles in the behavioral effects of drug abuse, in part through these mechanisms, by influencing neuroplasticity [21,22,23,24,25,26,27]. Using repeated-measures positron emission tomography (PET) imaging in vivo, our group has previously shown that cocaine significantly decreases class IIa HDAC expression-activity in the NAC and HC of male rats based on the amount of cocaine intake [25]. While studies are mixed, there is good evidence implicating class IIa HDACs in the behavioral effects of morphine [18,21,28]; however, the role of these epigenetic modulators in the behavioral effects of fentanyl is unknown.

To address this knowledge gap, we investigated fentanyl-induced behavioral sensitization in female and male rats and employed longitudinal PET imaging using 6-([^18^F]fluroacetamido)-1-hexanoicanilide ([^18^F]TFAHA) that targets class IIa HDAC expression-activity [25,29]. We hypothesized that both female and male rats will show significant fentanyl-induced LMA and behavioral sensitization and that females will display greater behavioral responses compared to males. Furthermore, we hypothesized that [^18^F]TFAHA PET imaging will show fentanyl-induced decreases in class IIa HDAC expression-activity in brain regions associated with reward and addiction based on previous research [25,30,31,32].

## 2. Materials and Methods

### 2.1. Subjects and Experimental Design

All experiments were conducted at Wayne State University (WSU) in Detroit, MI. Before initiating studies, the procedures were approved by WSU’s radiation safety program (Type A broad-scope license NRC) as well as the WSU Institutional Animal Care and Use Committee, and all procedures adhered to the *Guide for the Care and Use of Laboratory Animals* [33]. Rats were bred at Wayne State University and were heterozygotic for the Fos-LacZ transgene (Wistar-Tg(Fos-LacZ)1Ottc), although this transgene was not utilized in this study [34,35]. Adult female (n = 11, *m* = 196.4 g ± 2.6 *SEM*) and male (n = 11, *m =* 277.3 g ± 10.5 *SEM*) Wistar rats were used as part of this study. All animals were pair-housed by sex and condition in a temperature- and humidity-controlled vivarium, given food and water *ad libitum* in their home cages, and housed on a reversed 12 h light/dark cycle (lights on at 6 PM and off at 6 AM). All animals were handled and weighed daily throughout the study, and multiple small cohorts (3–6 rats), including both treatment groups (counterbalanced), were used to complete the study. Note that vaginal lavage samples were collected on days 0, 1, 14 and 28 from all female rats for later cytological characterization of the estrus phase, and male rats underwent a sham lavage using previously described procedures [13]. While these data were collected, the small number of subjects precluded the division of female data by estrus phases and the use of properly powered statistics to adequately analyze the data; therefore, the effects of the estrus cycle on fentanyl-induced outcomes have been omitted from analyses.

**Figure 1:**
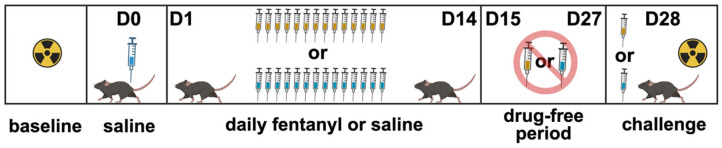
A schematic of the study design depicting days (D) of PET imaging (radiation symbol), locomotor activity assessment (running rat), drug administration period (saline control = blue syringe; fentanyl = orange syringe), drug-free period (“do not” symbol over syringes), and challenge (single syringe).

As shown in Figure 1, female and male rats were subjected to repeated fentanyl or saline administration followed by a drug-free period and a challenge drug exposure, along with pre- and post-drug assessments of LMA and PET imaging. About 1–2 weeks prior to the beginning of drug administration or behavioral assessment, all animals were subjected to baseline PET imaging in vivo using [^18^F]TFAHA, an HDAC class IIa-specific radiotracer. [^18^F]TFAHA is a highly selective substrate for class IIa HDACs and its breakdown product ([^18^F]-trifluoroacetate) gets trapped in the cells and its accumulation levels correspond to the HDACs’ expression and activity levels. On day 0 all animals received a subcutaneous (s.q.) saline (1 mL/kg) injection. However, from day 1 onwards they were randomly assigned to saline (male n = 6, female n = 5) or fentanyl (male n = 5, female n = 6) groups and received one s.q. injection of saline (1 mL/kg) or fentanyl (20 μg/kg) daily for 14 consecutive days (days 1–14). On days 2–13, animals were weighed, handled, and given a s.q. injection commensurate with their experimental group. On days 1 and 14, the animals were weighed, handled, given their injection and then placed into an open field to measure LMA (see Section 2.2 below). Following 14 days of daily drug administration, all animals were subjected to a drug-free period (i.e., a forced abstinence-like period) on days 15–27 in their home cages with handling and weighing but no injections. Finally, on day 28, following the drug-free period, they received a challenge injection (s.q.) of saline or fentanyl and all rats were again subjected to LMA assessment and then PET imaging with [^18^F]TFAHA in vivo.

### 2.2. Locomotor Activity (LMA) Assessment

All animals were subjected to LMA assessment for 30 min in an open field on days 0, 1, 14, and 28 following saline or fentanyl administration. The open field was a custom-made plexiglass behavioral chamber (70 × 30 cm) with removable wire covers as lids (Formtech Plastics, Oak Park, MI, USA). Animals were placed into these chambers, and their LMA was digitally recorded by overhead cameras and tracked using Ethovision XT 14 video tracking software (NOLDUS Information Technology, Wageningen, The Netherlands). Following completion of the behavioral tracking, animals were placed into their home cage. Distance traveled (m) was recorded to assess LMA, and behavioral sensitization was observed by increases during the drug administration days compared to day 0. Additionally, the percent change in LMA from day 0 to day 28 was calculated using the formula (((D28 − D0)/D0) × 100).

### 2.3. PET Imaging Using [^18^F]TFAHA

The radiosynthesis, formulation of [^18^F]TFAHA and PET imaging acquisitions were performed as previously described [25,29,36,37]. Approximately 11.1–18.5 MBq (300–500 μCi) of [^18^F]TFAHA in 1 mL sterile saline was administered intravenously on the lateral tail vein over the course of 1 min. The rats were then anesthetized (isoflurane 1.5–4% in oxygen) and secured on the heated (37 °C) bed of the Bruker Albira Si microPET R4 scanner (Siemens, Knoxville, TN, USA) to maintain body temperature. The brain was placed in the center of the field of view scanner. Static PET scans were acquired 1 h post-injection of the tracer. PET images were reconstructed using an ordered subset expectation–maximization method. Image registration, alignment, analysis and quantification were performed on the PMOD PFUS (version 4.4, Bruker, Zurich, Switzerland) wherein a digital rat brain atlas and template MRI of the rat brain were used to identify neuroanatomical markers for alignment. After co-registration, regions of interest (ROIs) were created in PMOD fusion tool (PFOS) to measure the uptake of [^18^F]TFAHA. The uptake was expressed as body weight (SUVbw)-normalized standard uptake values (SUV). Two investigators independently created ROI masks and quantified the uptake with an interrater variability of <5%. Anatomical localization was further validated by a series of landmarks including the harderian glands (robust and clear bilateral semicircular radiotracer accumulation), ear canals, and foramina. Following PET imaging at baseline, rats were quarantined for at least 3 days to allow for radioactive decay and allowed a further 4–11 days of rest prior to start of drug administration and LMA assessments. Rats were euthanized immediately following the PET imaging session on day 28 of the administration paradigm (e.g., challenge dose following drug-free period).

### 2.4. Statistical Analysis

The data were statistically analyzed and graphs were created using Prism 10.6.1 (GraphPad Software LLC, San Diego, CA, USA). Parametric analyses were used where assumptions of tests were met (e.g., 3-way ANOVA (all factors)); however, analyses following collapsing across non-significant factors (e.g., sex for PET imaging) not supported by the literature and appropriate assessments (2-way ANOVA) were used to further explore this data. Post hoc analyses were conducted using Tukey’s multiple comparison test and were evaluated using adjusted *p*-values to compensate for the number of comparisons being made. Non-parametric Mann–Whitney U analyses on % change were used following the non-significant ANOVAs for the HDAC analysis, where there were violations of test assumptions.

## 3. Results

### 3.1. Locomotor Activity and Behavioral Sensitization

**Figure 2:**
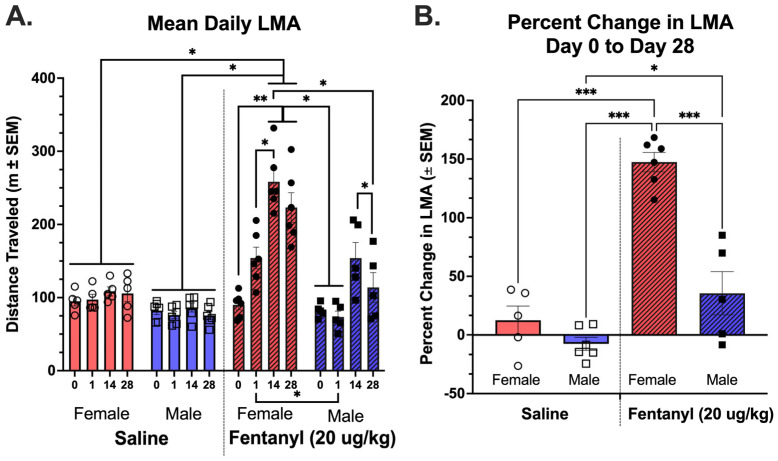
Panel (**A**) shows overall locomotor activity (LMA) for day 0 (saline injection), day 1 (first day of saline or fentanyl exposure), day 14 (final session of daily exposure), and day 28 (drug challenge day following drug-free period) separated by drug condition (bars with no pattern and open symbols = saline control, diagonal bars and filled symbols = fentanyl), group and sex (bars filled red and circle symbols = female, blue and square symbols= male). Panel (**B**) shows the percent change in LMA from day 0 to day 28. Significance between group means is shown as * *p* < 0.05, ** *p* < 0.01, or *** *p* < 0.001.

A three-way ANOVA demonstrated a significant three-way interaction between day (0, 1, 14, 28), sex (male/female) and drug (fentanyl/saline; F(2.125, 38.25) = 8.154, *p* < 0.001), with all two-way interactions (day × sex and day × drug, *p* < 0.01; Sex × Drug, *p* < 0.05) and main effects (*p* < 0.001) also being significant factors (see Supplementary Materials). Figure 2A demonstrates significant comparisons and an overall behavioral sensitization as measured by increased LMA from D0 during both the administration phase (D1 and 14; *p* < 0.01) as well as during a drug challenge phase (D28; *p* < 0.05). This finding is further contextualized by sex, with female rats receiving fentanyl demonstrating earlier (D1) and significantly greater sensitization overall (*p* < 0.05). Additionally, female rats receiving fentanyl demonstrated significant LMA increases on days 1, 14 and 28, whereas males only demonstrated this on day 14 and 28 but not day 1.

As shown in Figure 2B, we computed the percent change in LMA from day 0 (saline injection) to day 28 (drug challenge) to further contextualize behavioral sensitization. The one-way ANOVA on percent LMA change also demonstrated overall significance (F(3,18) = 40.10, *p* < 0.001). Post hoc analysis using Holm–Sidak’s multiple comparison test demonstrated that control females were significantly different from fentanyl females (*p* < 0.001) but not from control males (*p* = 0.337) or fentanyl males (*p* = 0.337). Control males were significantly different from both male (*p* < 0.05) and female (*p* < 0.001) fentanyl groups. Finally, the males exposed to fentanyl showed significantly less LMA compared to their female fentanyl counterparts (*p* < 0.001).

### 3.2. [^18^F]TFAHA PET Imaging

**Figure 3:**
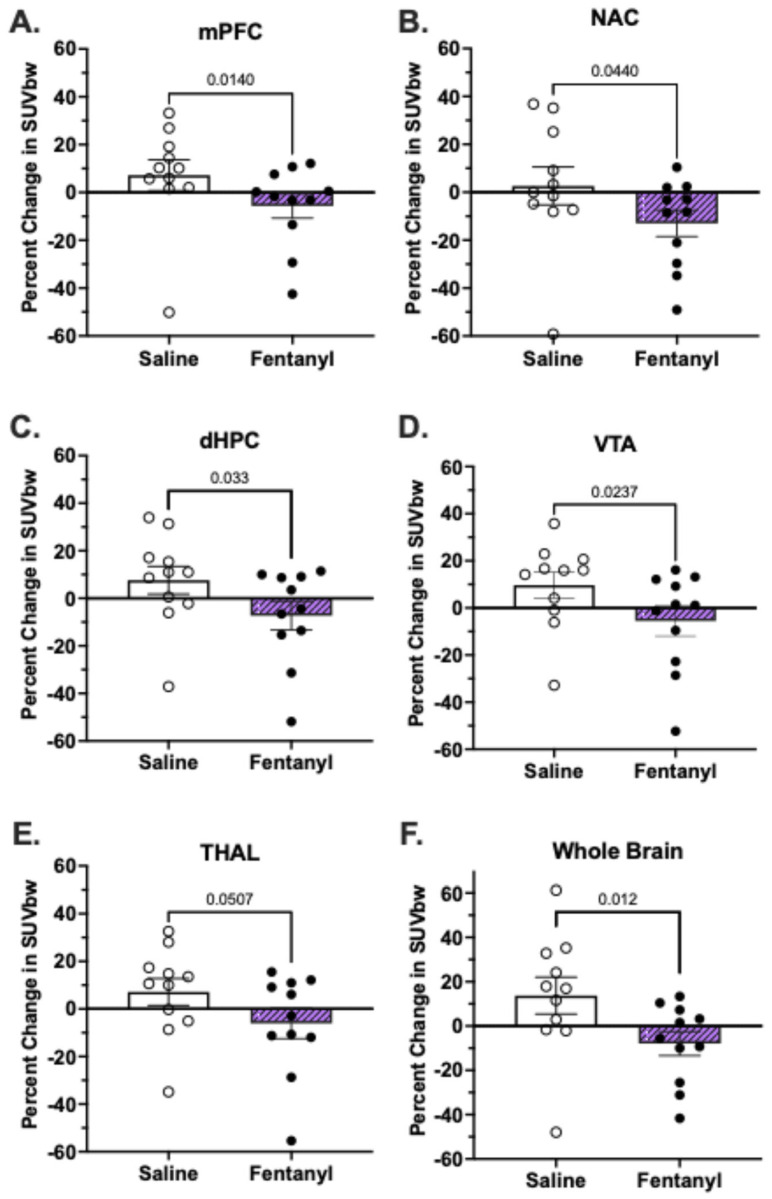
All panels depict a percent change in body weight-corrected standard uptake value (SUVbw) for regions of interest and whole brain. White bars and open symbols represent saline control and purple diagonal bars and filled symbols represent fentanyl treatment; collapsed by sex. Non-parametric Mann–Whitney U tests were conducted to compare means (all sample sizes were n = 11/group). Panel (**A**): medial prefrontal cortex (mPFC) actual difference (AD) = 11.84%, U = 27, *p* < 0.05. Panel (**B**): nucleus accumbens (NAC) AD = 8.2%, U = 34, *p* < 0.05. Panel (**C**): dorsal hippocampus (dHPC) AD = 15.39%, U = 27, *p* < 0.05. Panel (**D**): ventral tegmental area (VTA) AD = 15.97%, U = 30, *p* < 0.05. Panel (**E**): thalamus (THAL) AD = 10.62%, U = 35, *p* = 0.0507. Panel (**F**): whole Brain AD = 16.9%, U = 26, *p* < 0.05. All regions, including whole brain, showed significant (*p* < 0.05) decreases in SUVbw for the fentanyl-exposed group compared to saline with the exception of THAL (*p* = 0.0507).

Primary analyses using three-way ANOVA and two-way ANOVA were conducted (Table 1 shows the results from these analyses). Overall, analyses demonstrated non-significance (except sex for VTA, F(1,18) = 4.875, *p* = 0.041; see Supplementary Materials for ANOVA tables by region). However, these analyses did not meet key parametric assumptions, including a relatively small sample and uneven group sizes, which resulted in restricted range and non-normality. Given the violations and the distribution characteristics of the data, we conducted non-parametric analogs of these tests to ensure the quality of analyses and interpretation. Furthermore, due to these conditions and with consideration of the non-significant sex × drug condition interaction for every region (see Supplementary Materials), the sexes were collapsed (resulting in saline (control) and fentanyl (drug) groups) and the percent change from baseline to day 28 (pre and post) in SUVbw was calculated. This was intended to maximize the statistical power for detecting fentanyl-related effects. Mann–Whitney U non-parametric analysis for all of the regions (except for a trending result for thalamus (*p* = 0.0507)) demonstrated a significant decrease in SUV for fentanyl compared to the saline (*p* < 0.05; see Table 1 and Figure 3 below for results).

## 4. Discussion

This study investigated the effect of repeated fentanyl exposure followed by an abstinence-like, drug-free period on the ensuing behavioral sensitization. Additionally, the underlying epigenetic changes marked specifically by changes in class IIa HDAC expression-activity were investigated. Our results support that repeated fentanyl exposure induces behavioral sensitization characterized by increases in open-field LMA and appears to reduce class IIa HDAC expression-activity in the whole brain, including reward-related brain regions. Finally, sex differences in locomotor sensitization were observed as hypothesized, yet sex was not a factor in the reduced expression-activity of class IIa HDACs in the brain.

Behavioral sensitization in humans, also known as reverse tolerance, is manifested as increased psychomotor effects, including heightened alertness, focus, and enhanced eye blink rate [12]. Human studies that have assessed opioid-induced behavioral sensitization, including the effects of fentanyl, are lacking. However, studies investigating repeated low-dose amphetamine show enhanced characteristics of human behavioral sensitization such as increased motor activity, energy level, mood, and talkativeness (i.e., rate and amount of speech) [38,39,40,41].

The behavioral results of this study confirm our previous data showing profound behavioral sensitization in females compared to males [13]. For instance, in this study, females were more sensitive to a single dose of fentanyl as shown by elevated LMA on day 1 compared to their male counterparts. Furthermore, females displayed a robust increase in LMA following repeated fentanyl administration for 14 days in this study in line with our previous work using a shorter 7-day repeated fentanyl administration period [13]. Beyond the LMA sensitization, our previous study also found that fentanyl enhanced contextual reward behavior as evidenced by a marked conditioned place preference (CPP) in both females and males. Interestingly, though, males showed CPP in response to a 4 μg/kg dose, while females only displayed CPP to a higher dose (32 μg/kg), suggesting distinct neuroadaptations between the sexes in the rewarding and sensitizing effects of fentanyl [13]. Integration of these findings is challenging and will require further exploration, as the interpretation of fentanyl being aversive at lower doses in females is not well supported in the literature. Distinguishing between the appetitive/rewarding and LMA-sensitizing properties may help to clarify the dose–response differences that we saw in our investigations and in the literature and may lead to differential treatment paradigms for human users.

Our current finding of females responding with increased LMA after a single exposure is already in line with sex differences in substance use behaviors seen in humans. For example, females show more telescoping use patterns (i.e., faster progression from initial use to meeting diagnostic criteria) compared to their male counterparts, resulting in more serious consequences, including withdrawal and craving intensities, despite shorter durations of use [42,43]. Exploring sex differences in animal studies provides a translational approach to model observations in humans to understand mechanisms and develop novel pharmacotherapeutics.

In addition to the behavioral effects of fentanyl, our study demonstrated significant epigenetic effects in the brain of both sexes. We observed the hypothesized decrease in class IIa HDAC expression-activity in reward-related brain regions, including the mPFC, NAC, dHC and VTA. Interestingly, though, we found a whole-brain effect and a trending effect in the thalamus, which is a brain structure not directly involved with reward processing, suggesting the changes in class IIa HDACs may not underlie the long-lasting sensitizing effects of fentanyl on LMA. Furthermore, the disconnect between brain and behavior is supported by the lack of sex differences in class IIa HDAC-decreased expression-activity despite observing robust dimorphic behavioral sensitization (i.e., females are more pronounced than males).

Similar to the decrease in class IIa HDAC expression-activity caused by fentanyl in this study, we previously found that high cocaine intake significantly decreases class IIa HDAC expression-activity in NAC, while low cocaine intake significantly decreases expression-activity in dHPC of male rats using PET imaging with [^18^F]TFAHA [25]. However, few studies have explored the role of fentanyl and other opioids in modulating class IIa HDACs and related epigenetic changes, despite the potential insight to be gained. In a study investigating lung cancer, fentanyl inhibited the viability and invasion of cancerous cells by reducing levels of HDAC5 [44]. More closely aligned with this study, Anderson et al., [45] revealed that HDAC5 overexpression in NAC suppressed context-associated and reinstated heroin-seeking behavior. Several other studies have used pan-HDAC inhibitors to implicate HDACs in the rewarding effects of opioids. For example, Saberian et al., [46] found that administration of pan-HDAC inhibitor suberoylanilide hydroxamic acid (SAHA) in animals treated with morphine showed increased gene expression of ΔFosB in reward centers such as NAC and mPFC. And other investigations have similarly demonstrated that pan-HDAC inhibition can attenuate morphine-induced conditioned place preference and withdrawal symptoms [46,47,48,49,50]. Other investigations have explored substances of abuse including downregulation of HDAC4/7 mRNA expression in NAC in response to methamphetamine [30], while there is evidence of increases in HDAC4 (and HDACs 5 and 7 under some conditions) in skeletal muscle/myoblast models [51]. However, the research is mixed; for example, pan-HDAC inhibition prevents morphine tolerance in normal mice by inhibiting pro-inflammatory marker IL6 and upregulating MOR in neurons [52]. The current research on the epigenetic effects of fentanyl and other opioids, including the data herein, implicates HDACs and specifically class IIa HDACs. An equal interpretation for our current findings could be that the change in HDAC activity results from fentanyl administration; however, it is not involved with the behavioral change. Additionally, since the current investigation observed a relatively short timescale, these changes could be long-lasting or relatively transient in nature. Gaining more information about these two factors could help us understand the role that HDAC activity plays in response to fentanyl. Considerably more research is needed to understand the epigenetic landscape, including studies that consider sex as a biological variable.

To that end, there are a few limitations in the current study that future investigations should consider when replicating and extending this study. First, the current study does not directly measure histone acetylation, chromatin state, HDAC mRNA/protein expression, or subcellular localization (e.g., nuclear vs. cytoplasm). The PET-based imaging provides a good indication that there is an induction of epigenetic change, but this needs to be further expanded and supported by direct molecular measurements. Each of these methodologies would provide much more explicit molecular measurements, validate the findings from this paper, and expand our understanding of the epigenetic changes induced by fentanyl. Secondly, finding sex differences in behavioral data but not replicating these within the molecular findings was surprising. The move from a parametric three-way ANOVA to a non-parametric Mann–Whitney U, due to not finding a significant sex x drug condition interaction, limits the interpretation of sex differences for our molecular findings. Future investigations should potentially include slightly larger groups (e.g., 8–10 animals) to better power their studies to observe what appears to be a smaller effect size for sex differences if they exist.

Our findings represent the first steps to encapsulate behavioral and sex differences related to the epigenetic effects of fentanyl in the brain. Although we demonstrated a decline in class IIa HDACs in the reward circuitry, we observed a disconnect in class IIa HDACs in the brain with the behavioral sex effects, and we found that lower class IIa HDACs were also found in the whole brain, rather than being circuit-specific. These observations lead us to speculate that class IIa HDACs play a yet-to-be-determined role in the behavioral response to fentanyl.

## Figures and Tables

**Table 1:** The baseline and day 28 (D28) means (+/−SEM; n) of body weight-corrected standard uptake value (SUVbw) by region for the individual sexes (colored cells; red = female, blue = male) and the total (sex collapsed; white cells) by group and the percent change from baseline to day 28. “†” indicates that the significance value comes from multiple comparison tests following a 3-way ANOVA on the raw data. “††” indicates that the significance value comes from multiple comparisons following a 2-way ANOVA comparing percent change. “‡” indicates that the significance comes from a non-parametric Mann–Whitney U. If the value was non-significant but was below *p* = 0.1 (e.g., trending), the *p*-value was provided alongside a N.S. designation.

Brain Region	Sample	Saline Mean SUVbw (S.E.M.; n)				Fentanyl Mean SUVbw (S.E.M.; n)				% Change in SUVbw Saline vs. Fentanyl
		Baseline	D28	Significance Raw Pre to Post	Average % Change	Baseline	D28	Significance Raw Pre to Post	Average % Change	
**mPFC**	Female	0.56 (0.04;5)	0.54 (0.08;5)	N.S. ^†^	−2.8%	0.55 (0.02;6)	0.52 (0.03;6)	N.S. ^†^	−5.3%	N.S. ^††^
	Male	0.50 (0.02;6)	0.58 (0.01;6)	N.S. ^†^	15.0%	0.53 (0.02;5)	0.49 (0.04;5)	N.S. ^†^	−8.0%	0.071; N.S. ^††^
	Total	0.53 (0.02;11)	0.56 (0.04;11)	N.S. ^††^	6.4%	0.54 (0.02;11)	0.50 (0.02;11)	N.S. ^††^	−6.5%	*p* = 0.014 ^‡^
**NAC**	Female	0.62 (0.05;5)	0.56 (0.08;5)	N.S. ^†^	−10.0%	0.60 (0.02;5)	0.53 (0.04;5)	N.S. ^†^	−12.0%	N.S. ^††^
	Male	0.56 (0.02;6)	0.62 (0.01;6)	N.S. ^†^	10.2%	0.56 (0.02;5)	0.47 (0.05;5)	N.S. ^†^	−16.0%	0.079; N.S. ^††^
	Total	0.59 (0.02;11)	0.59 (0.04;11)	N.S. ^††^	0.5%	0.59 (0.02;11)	0.50 (0.03;11)	*p* = 0.045 ^††^	−13.8%	*p* = 0.044 ^‡^
**dHPC**	Female	0.47 (0.04;5)	0.48 (0.08;5)	N.S. ^†^	1.7%	0.49 (0.02;6)	0.46 (0.04;6)	N.S. ^†^	−6.0%	N.S. ^††^
	Male	0.41 (0.02;6)	0.47 (0.02;6)	N.S. ^†^	13.9%	0.44 (0.02;5)	0.40 (0.04;5)	N.S. ^†^	−9.8%	0.072; N.S. ^††^
	Total	0.44 (0.02;11)	0.48 (0.04;11)	N.S. ^††^	7.9%	0.46 (0.01;11)	0.43 (00.3;11)	N.S. ^††^	−7.6%	*p* = 0.033 ^‡^
**VTA**	Female	0.51 (0.05;5)	0.53 (0.09;5)	N.S. ^†^	4.2%	0.53 (0.02;6)	0.49 (0.02;6)	N.S. ^†^	−6.4%	N.S. ^††^
	Male	0.42 (0.02;6)	0.48 (0.02;6)	N.S. ^†^	15.7%	0.44 (0.02;5)	0.41 (0.05;5)	N.S. ^†^	−6.1%	N.S. ^††^
	Total	0.46 (0.02;11)	0.51 (0.04;11)	N.S. ^††^	9.9%	0.49 (0.02;11)	0.46 (0.03;11)	N.S. ^††^	−6.3%	*p* = 0.024 ^‡^
**THAL**	Female	0.48 (0.04;5)	0.50 (0.08;5)	N.S. ^†^	4.3%	0.49 (0.02;6)	0.48 (0.04;6)	N.S. ^†^	−2.9%	N.S. ^††^
	Male	0.43 (0.02;6)	0.47 (0.02;6)	N.S. ^†^	10.5%	0.44 (0.02;5)	0.39 (0.04;5)	N.S. ^†^	11.7%	N.S. ^††^
	Total	0.45 (0.02;11)	0.49 (0.04;11)	N.S. ^††^	7.5%	0.47 (0.01;11)	0.44 (0.03;11)	N.S. ^††^	−6.7%	*p* = 0.051 ^‡^
**Whole Brain**	Female	0.53 (0.04;5)	0.52 (0.08;6)	N.S. ^†^	−2.1%	0.53 (0.02;6)	0.49 (0.04;6)	N.S. ^†^	−9.2%	N.S. ^††^
	Male	0.46 (0.02;6)	0.58 (0.04;6)	N.S. ^†^	27.1%	0.47 (0.01;5)	0.44 (0.05;5)	N.S. ^†^	−6.5%	*p* = 0.018
	Total	0.49 (0.02;11)	0.55 (0.04;11)	N.S. ^††^	12.7%	0.50 (0.01;11)	0.46 (0.03;11)	N.S. ^††^	−8.1%	*p* = 0.012 ^‡^

## Data Availability

The data presented in this study are available on request from the corresponding author (S.A.P.).

## Supplementary Materials

The following supporting information can be downloaded at: https://www.mdpi.com/article/10.3390/brainsci16070684/s1.

## Abbreviations

The following abbreviations are used in this manuscript:

HDAC	Histone Deacetylase
PET	Positron Emission Tomography
LMA	Locomotor Activity
