# Supplementary material for: Fentanyl Induces Behavioral Sensitization and Decreases Class IIa HDAC Expression-Activity in Brain as Measured by [18F]TFAHA PET Imaging in Female and Male Rats
Source: Brain Sci. 2026 Jun 29;16(7):684. doi: 10.3390/brainsci16070684 (PMC13407376; doi:10.3390/brainsci16070684)
Supplement: Supplementary file 1 [file brainsci-16-00684-s001.zip › brainsci-4322663-supplementary.pdf]

LMA (Behavioral Analysis)

ANOVA table	SS	DF	MS	F (DFn, DFd)	P value
Day	55264	3	18421	F (2.125, 38.25) = 66.63	P<0.0001
Sex	60798	1	60798	F (1, 18) = 26.60	P<0.0001
Drug	51440	1	51440	F (1, 18) = 22.51	P=0.0002
Day x Sex	40696	3	13565	F (2.125, 38.25) = 49.07	P<0.0001
Day x Drug	11588	3	3863	F (2.125, 38.25) = 13.97	P<0.0001
Sex x Drug	15922	1	15922	F (1, 18) = 6.966	P=0.0167
Day x Sex x Drug	6763	3	2254	F (2.125, 38.25) = 8.154	P=0.0009
Subject	41141	18	2286		
Residual	14930	54	276.5		

HDAC (Molecular Analysis)

mPFC

ANOVA table	SS	DF	MS	F (DFn, DFd)	P value
Pre/post	9E-05	1	9E-05	F (1, 18) = 0.01469	P=0.9049
Sex	0.0026	1	0.0026	F (1, 18) = 0.2930	P=0.5950
Drug Condition	0.0077	1	0.0077	F (1, 18) = 0.8552	P=0.3673
Pre/post x Sex	0.0041	1	0.0041	F (1, 18) = 0.7061	P=0.4118
Pre/post x Drug Condition	0.0117	1	0.0117	F (1, 18) = 1.996	P=0.1747
Sex x Drug Condition	0.0005	1	0.0005	F (1, 18) = 0.05289	P=0.8207
Pre/post x Sex x Drug Condition	0.0075	1	0.0075	F (1, 18) = 1.282	P=0.2723
Subject	0.1615	18	0.009		
Residual	0.1055	18	0.0059		

NAC

ANOVA table	SS	DF	MS	F (DFn, DFd)	P value
Pre/post	0.0191	1	0.0191	F (1, 18) = 1.750	P=0.2024
Sex	0.0069	1	0.0069	F (1, 18) = 0.9685	P=0.3381
Drug condition	0.0241	1	0.0241	F (1, 18) = 3.398	P=0.0818
Pre/post x Sex	0.0071	1	0.0071	F (1, 18) = 0.6547	P=0.4290
Pre/post x Drug Condition	0.017	1	0.017	F (1, 18) = 1.559	P=0.2279
Sex x Drug condition	0.0078	1	0.0078	F (1, 18) = 1.092	P=0.3098
Pre/post x Sex x Drug Condition	0.0128	1	0.0128	F (1, 18) = 1.174	P=0.2929
Subject	0.1279	18	0.0071		
Residual	0.1963	18	0.0109		

dHPC

ANOVA table	SS	DF	MS	F (DFn, DFd)	P value
Pre-Post	3E-05	1	3E-05	F (1, 18) = 0.007248	P=.933
Sex	0.0227	1	0.0227	F (1, 18) = 1.879	P=.187
Drug Condition	0.0027	1	0.0027	F (1, 18) = 0.2217	P=.643
Pre-Post x Sex	0.0009	1	0.0009	F (1, 18) = 0.2055	P=.656
Pre-Post x Drug Condition	0.0129	1	0.0129	F (1, 18) = 3.031	P=.099
Sex x Drug Condition	0.0009	1	0.0009	F (1, 18) = 0.07439	P=.788
Pre-Post x Sex x Drug Condition	0.0027	1	0.0027	F (1, 18) = 0.6273	P=.439
Subject	0.2171	18	0.0121		
Residual	0.0765	18	0.0042		

VTA

ANOVA table	SS	DF	MS	F (DFn, DFd)	P value
Pre/post	0.0005	1	0.0005	F (1, 18) = 0.09767	P=0.7582
Sex	0.0639	1	0.0639	F (1, 18) = 4.875	P=0.0405
Condition	0.0042	1	0.0042	F (1, 18) = 0.3181	P=0.5797
Pre/post x Sex	0.0018	1	0.0018	F (1, 18) = 0.3560	P=0.5582
Pre/post x Condition	0.0149	1	0.0149	F (1, 18) = 2.989	P=0.1010
Sex x Condition	0.0007	1	0.0007	F (1, 18) = 0.05112	P=0.8237
Pre/post x Sex x Condition	0.001	1	0.001	F (1, 18) = 0.1968	P=0.6626
Subject	0.2358	18	0.0131		
Residual	0.0896	18	0.005		

Thal

ANOVA table	SS	DF	MS	F (DFn, DFd)	P value
Pre/post	2E-07	1	2E-07	F (1, 18) = 3.950e-005	P=0.9951
Sex	0.0291	1	0.0291	F (1, 18) = 2.428	P=0.1366
Drug Condition	0.0045	1	0.0045	F (1, 18) = 0.3728	P=0.5491
Pre/post x Sex	0.0001	1	0.0001	F (1, 18) = 0.02336	P=0.8802
Pre/post x Drug Condition	0.0119	1	0.0119	F (1, 18) = 2.363	P=0.1417
Sex x Drug Condition	0.0018	1	0.0018	F (1, 18) = 0.1542	P=0.6991
Pre/post x Sex x Drug Condition	0.0026	1	0.0026	F (1, 18) = 0.5197	P=0.4802
Subject	0.2156	18	0.012		
Residual	0.0906	18	0.005		

Whole Brain

ANOVA table	SS	DF	MS	F (DFn, DFd)	P value
Pre/Post	0.0007	1	0.0007	F (1, 18) = 0.1278	P=.725
Sex	0.0119	1	0.0119	F (1, 18) = 1.021	P=.326
Drug Condition	0.0182	1	0.0182	F (1, 18) = 1.571	P=.226
Pre/Post x Sex	0.0161	1	0.0161	F (1, 18) = 2.789	P=.112
Pre/Post x Drug Condition	0.0251	1	0.0251	F (1, 18) = 4.344	P=.052
Sex x Drug Condition	0.0074	1	0.0074	F (1, 18) = 0.6342	P=.436
Pre/Post x Sex x Drug Condition	0.0091	1	0.0091	F (1, 18) = 1.577	P=.225
Subject	0.2088	18	0.0116		
Residual	0.1038	18	0.0058		
